# Sentinel Node Biopsy for Endometrial Cancer by Retroperitoneal Transvaginal Natural Orifice Transluminal Endoscopic Surgery: A Preliminary Study

**DOI:** 10.3389/fsurg.2022.907548

**Published:** 2022-05-09

**Authors:** Daniela Huber, Yannick Hurni

**Affiliations:** ^1^ Department of Gynecology and Obstetrics, Valais Hospital, Sion, Switzerland; ^2^ Department of Pediatrics, Gynecology and Obstetrics, Geneva University Hospitals, Geneva, Switzerland

**Keywords:** sentinel node, indocyanine green (ICG), endometrial cancer, natural orifice transluminal endoscopic surgery (NOTES), surgical staging

## Abstract

**Objective:**

The primary objective was to determine the intraoperative and postoperative surgical complications of sentinel lymph node biopsy (SLNB) by retroperitoneal transvaginal natural orifice transluminal endoscopic surgery (vNOTES). The secondary objective was to assess the feasibility of this surgical technique.

**Methods:**

This was a descriptive study realized in a non-university hospital in Switzerland. Seven patients with endometrial cancer or endometrial complex atypical hyperplasia underwent surgical staging with SLNB by retroperitoneal vNOTES using an indocyanine green-based near-infrared fluorescence imaging technique (October 2021–February 2022).

**Results:**

The median operative time was 113 (81–211) minutes. The median estimated blood loss was 20 (20–400) mL. The overall bilateral detection rate was 100% (7/7). Upon histopathological examination, 5 patients presented an endometrial adenocarcinoma, and we found endometrial complex atypical hyperplasia in 2 cases. We successfully completed all procedures without significant intraoperative complications, but 1 case required conversion to conventional laparoscopy. The median postoperative stay was 2 (2–4) days, and we observed no postoperative complications during this period. We observed 1 case of postoperative deep vein thrombosis and an asymptomatic vaginal vault hematoma in the same patient.

**Conclusions:**

Our preliminary study suggests that retroperitoneal vNOTES could be a feasible, safe, and valuable approach to perform SLNB in endometrial cancer. However, strong evidence of its feasibility, the effective benefits, and the long-term oncological outcomes is needed before expanding the use of vNOTES in endometrial cancer outside study settings.

## Introduction

Sentinel lymph node biopsy (SLNB) has been increasingly used as a method to identify lymph node metastases in gynecological malignancies while minimizing the complications associated with extensive lymphadenectomy (1). This technique has emerged as an accurate and feasible tool for the surgical staging of early-stage endometrial cancers (1). Compared to complete lymphadenectomy, SLNB seems to improve surgical outcomes while preserving reliable surgical staging results (2). Surgical staging and SLNBs are generally performed using minimally invasive techniques, such as conventional laparoscopy or robotic-assisted laparoscopy (1–5). To reduce the morbidity related to transabdominal surgeries further, endometrial cancer staging with transvaginal natural orifice transluminal endoscopic surgery (vNOTES) techniques have been introduced (6, 7). vNOTES allows for the performance of complete surgical staging with hysterectomy, salpingo-oophorectomy, SLNB, and eventually pelvic and para-aortic lymphadenectomy (6, 8–10). In a recent study, vNOTES endometrial cancer staging with SLNB appeared comparable to conventional laparoscopy in terms of safety and efficacy while avoiding visible scars and presenting a shorter hospital stay and fewer perioperative complications (9). SLNB by vNOTES can be performed with a transperitoneal (9) or retroperitoneal approach (8). Although the retroperitoneal approach seems to provide better exposure of the pelvic lymph nodes, this technique has been reported only twice in the literature (8, 11). In the present study, we report our initial experience using SLNB with a retroperitoneal vNOTES approach for the surgical staging of endometrial cancer.

## Materials and Methods

### Patient Selection and Data Collection

We retrospectively reviewed patients with endometrial cancer or premalignancy who had undergone surgical staging with SLNB with retroperitoneal vNOTES at our institution between October 2021 and February 2022. All patients gave written informed consent, and the same surgeon (D.H.) performed all interventions at Valais Hospital in Sion (Switzerland). We received approval from the local ethical committee (CER-VD), with registration number 2021-02346.

The patients were selected according to the following criteria: (1) low-grade (grade 1 or 2) endometrial adenocarcinoma or complex atypical hyperplasia (CAH) on preoperative endometrial biopsy with lesions confined to the uterus (transvaginal ultrasound and/or magnetic resonance imaging); (2) no preoperative evidence of intraperitoneal, lymphatic, or distant metastases; (3) preoperative multidisciplinary tumor board discussion with the indication to perform surgical staging with SLNB; and (4) SLNB performed by retroperitoneal vNOTES.

Demographic features, as well as clinical and perioperative information, were retrospectively collected from a chart review. Intraoperative complications included bleeding requiring transfusion and procedural iatrogenic organ damage. Postoperative complications were graded using the Clavien-Dindo classification (CD) (12) and included complications that occurred up to 8 weeks after surgery.

### Surgical Technique

Patients were placed in dorsal lithotomy position under general anesthesia and received prophylactic intravenous antibiotics with cefuroxime 1.5 g and metronidazole 500 mg. A total of 2 mL of indocyanine green (ICG) solution at a 1.25 mg/mL concentration was injected into the cervix at the 3 and 9 o’clock positions. During SLNBs, the operating table was maintained horizontal without Trendelenburg tilt. Access to the pelvic retroperitoneal space was achieved through a 2-cm incision in the lateral vaginal fornix, providing access to the obturator fossa. A 7 cm GelPoint V-Path Transvaginal Access Platform (Applied Medical, Rancho Santa Margarita, CA, USA) was used as a vNOTES port, and CO2 was insufflated to expand the retroperitoneal space to a pressure of 10–15 mmHg. We used 3 trocars to insert a 10-mm rigid 30° camera, 5-mm instruments such as Johan and bipolar graspers, and a Caiman® articulating sealing device. Careful dissection was performed from the caudal part of the obturator fossa to develop the pelvic retroperitoneal space. The obturator nerve, the ureter, and the external and internal iliac vessels were identified. The pelvic retroperitoneal space was inspected for ICG uptake by lymph nodes under visual guidance using a near-infrared fluorescent optic device (ENDOCAM Logic 4k, Richard Wolf GmbH, Knittlingen, Germany). The sentinel lymph nodes (SLNs) were identified and separated from the surrounding tissues by careful dissection with the Caiman® device. In addition to ICG-fluorescence-positive SLNs, any other suspicious lymph nodes were excised and removed. The same procedure was subsequently performed contralaterally. The surgical technique for SLNB is demonstrated in Supplementary Video S1. Subsequently, the intervention was completed with a vaginally assisted NOTES hysterectomy with bilateral salpingectomy, oophorectomy, or salpingo-oophorectomy, as appropriate. All specimens were extracted transvaginally under direct endoscopic vision. At the end of the procedure, we closed the colpotomy with a running suture using a Stratafix Spiral PDS 0. Clindamycin vaginal cream was administered once a day for the first 7 postoperative days.

### Statistical Analyses

The primary objective was to determine the intraoperative and postoperative surgical complications. The secondary objective was to assess this surgical technique’s feasibility by evaluating the SLNs-detection rate and the conversion rate to conventional laparoscopy or laparotomy. We present continuous variables by their median with minimum and maximum values, and categorical variables as numbers and percentages.

## Results

Seven patients with endometrial malignancies or premalignancies underwent SLNB by vNOTES between October 2021 and February 2022. Table 1 summarizes the patients’ demographic and perioperative characteristics. The median age was 68 (45–83) years, and the median body mass index was 26.4 (22.3–44.6) kg/m^2^. Five patients were multiparous, while two presented no previous vaginal delivery. Preoperative endometrial biopsies showed endometrial adenocarcinoma in 4 patients and CAH in 3 patients.

**Table 1:** Demographics and perioperative characteristics.

Patient no.	Age (y)	BMI (Kg/m^2^)	Parity	Previous abdominal/pelvic surgery	Preoperative diagnosis (stage)	Type of intervention	Operative time (min)	Blood loss (mL)	Intraoperative complications	Stay (d)	Postoperative complications
1	64	22.4	2	Laparoscopic tubal sterilization	Endometrial endometrioid adenocarcinoma (T2 N0 M0)	Retroperitoneal vNOTES pelvic SLNB, VANH with bilateral salpingo-oophorectomy	113	20	None	2	None
2	83	22.3	2	None	Endometrial endometrioid adenocarcinoma (T1b N0 M0)	Retroperitoneal vNOTES pelvic SLNB, VANH with bilateral salpingo-oophorectomy	211	400	Conversion to conventional laparoscopy for bleeding from the left obturator fossa	2	None
3	68	32.0	2	None	Endometrial endometrioid adenocarcinoma (T1b N0 M0)	Retroperitoneal vNOTES pelvic SLNB, VANH with bilateral salpingo-oophorectomy	88	20	None	2	DVT on the 20^th^ postoperative day (CD II), and subsequent asymptomatic vaginal vault hematoma (CD I)
4	77	29.6	6	Laparotomic bilateral salpingectomy (sterilization) and laparoscopic cholecystectomy	Endometrial endometrioid adenocarcinoma (T1b N0 M0)	Retroperitoneal vNOTES pelvic SLNB, VANH with bilateral oophorectomy	143	20	None	4	None
5	45	24.0	2	None	Endometrial complex atypical hyperplasia	Retroperitoneal vNOTES pelvic SLNB, VANH with bilateral salpingectomy	81	20	Subcutaneous emphysema with spontaneous resolution	2	None
6	62	44.6	2	Two cesarean sections	Endometrial complex atypical hyperplasia	Retroperitoneal vNOTES pelvic SLNB, VALH with bilateral salpingo-oophorectomy	146	20	None	2	None
7	76	26.4	0	Laparoscopic appendectomy	Endometrial complex atypical hyperplasia	Retroperitoneal vNOTES pelvic SLNB, VALH with bilateral salpingo-oophorectomy	100	50	None	2	None

*vNOTES*, *Transvaginal Natural Orifice Transluminal Endoscopic Surgery; SLNB*, *Sentinel Lymph Node Biopsy; VANH*, *Vaginally Assisted NOTES hysterectomy; CD*, *Clavien-Dindo classification; DVT*, *Deep vein thrombosis.*

All patients underwent bilateral SLNB followed by a vaginally assisted NOTES hysterectomy. In addition, bilateral salpingo-oophorectomy was performed in 6 patients, while ovaries were spared in patient 5 (CAH in premenopausal). The median operative time, calculated from the first incision to complete closure, was 113 (81–211) minutes. The median estimated blood loss was 20 (20–400) mL. No significant intraoperative complications occurred, and no patient required a blood transfusion. During CO2 insufflation to expand the right pelvic retroperitoneal space, patient 5 developed subcutaneous emphysema on the right hemithorax without hypercarbia, and the surgery could be completed without any ventilatory problem. Pneumothorax, pneumomediastinum, and pleural effusion were excluded, with spontaneous resolution of the emphysema. Conversion to conventional laparoscopy was necessary to complete the intervention in patient 2. After successful bilateral pelvic SLNB, the patient presented slight continuous bleeding from the left obturator fossa, and the surgeon decided to convert to conventional laparoscopy. Bleeding from a left deep vaginal vein was observed, and successful hemostasis was performed.

The median postoperative stay was 2 (2–4) days, and we observed no postoperative complications during hospitalizations. Patient 3 presented a deep vein thrombosis (DVT) of the right common femoral, superficial femoral, and popliteal veins on the 20th postoperative day (CD grade II). The patient was treated with rivaroxaban 20 mg/day for 6 months. On the 30th postoperative day, the same patient was seen for routine postoperative control and presented an asymptomatic vaginal vault hematoma with a greater diameter of 8 cm on ultrasound examination. This hematoma was not present at the time of the DVT diagnosis. No interventions were performed, and the patient could follow the adjuvant treatment as planned (CD grade I).

Table 2 shows the characteristics of SLNs and histopathology. The SLN bilateral detection rate was 100% (7/7). We detected 13 SLNs on the left side and 9 on the right side. The distribution of the 22 SLNs by anatomic location was as follows: external iliac (17, 77.3%) and common iliac (5, 22.7%). We observed no lymph node metastases, but we found isolated tumor cells in 1 SLN in patient 2. Of the 3 cases with endometrial CAH, 2 presented no endometrial cancer, but we found adenocarcinoma in patient 7. We confirmed the 4 cases with a preoperative diagnosis of endometrial adenocarcinoma at definite histology. Endometrial cancer stages were pT1bN0 in 3 cases, pT2N0 in 1 case, and pT3bN0 in 1 case (microscopic right parametrial involvement). Table 2 shows oncological adjuvant therapies.

**Table 2:** Characteristics of sentinel lymph nodes, histopathology, and oncological adjuvant therapy.

Patient no.	Location of SLNs	Number of SLNs			SLNs metastases	Histopathology			Oncological adjuvant therapy
		Total	Left	Right		Histology	Stage	Grade	
1	Left external iliac; right external iliac	3	2	1	0/3	Endometrial endometrioid adenocarcinoma	pT2 N0(i-)(sn)(0/3) L0 V0 Pn0	1	ERT and VBT
2	Left external iliac; right external iliac	2	1	1	0/2 ITC (right SLN)	Endometrial endometrioid (90%) and mucinous (10%) adenocarcinoma	pT1b N0(i+)(sn)(0/2) L1 V1 Pn0	1–2	VBT
3	Left common iliac; right common iliac	4	3	1	0/4	Endometrial endometrioid adenocarcinoma	pT1b N0(i-)(sn)(0/4) L0 V0 Pn0	1	VBT
4	Left external iliac; right external iliac	6	4	2	0/6	Endometrial endometrioid adenocarcinoma	pT3b N0(i-)(sn)(0/6) L1 V0 Pn1	2	ERT, CT^a^ and VBT
5	Left external iliac; right common iliac	2	1	1	0/2	Endometrial complex atypical hyperplasia	–	–	–
6	Left external iliac; right external iliac	3	1	2	0/3	Endometrial complex atypical hyperplasia	–	–	–
7	Left external iliac; right external iliac	2	1	1	0/2	Endometrial endometrioid adenocarcinoma	pT1b N0(i-)(sn)(0/2) L0 V0 Pn0	1	VBT

*SLN*, *Sentinel Lymph Node; ERT*, *External Radiotherapy; VBT*, *Vaginal Brachytherapy; ITC*, *Isolated Tumor Cells; CT*, *Chemotherapy.*

^a^ *A concomitant radio-chemotherapy and subsequent chemotherapy were initially proposed, but in accordance with the patient’s frailty and wishes, we finally performed radiotherapy only*.

## Discussion

We report our initial experience with SLNB performed with a retroperitoneal vNOTES approach for the surgical staging of endometrial cancer. We were able to complete the intervention with bilateral SLNBs in all patients. Although this technique appeared feasible without significant intraoperative complications, conversion to conventional laparoscopy was required to manage persistent pelvic bleeding in 1 case at the beginning of our series. Conversion to laparoscopy was reported only once in previous studies reporting SLNB by vNOTES (7–11, 13), and it generally seems rarely required during gynecological vNOTES procedures (14). Although our case seemed associated with initial inexperience, proper hemostasis of deep vaginal vessels could be challenging with vNOTES due to the difficulty in accessing this anatomical area. In addition, we observed a case of intraoperative self-limited subcutaneous emphysema. Subcutaneous emphysema is frequently observed following laparoscopic procedures (15). Although usually harmless, cases of life-threatening complications have been reported, such as pneumothorax, pneumomediastinum, and pneumopericardium (16). Because subcutaneous emphysema seems more frequently associated with retroperitoneal procedures (17), particular attention should be paid in future studies focused on retroperitoneal vNOTES procedures.

We observed no postoperative complications in all patients except one who presented a DVT with a subsequent asymptomatic vaginal vault hematoma probably related to the anticoagulant therapy. In a large observational study, postoperative hematoma following vNOTES hysterectomy was observed in 11 of 750 patients (1.5%), with surgical drainage required in 6 patients (0.8%) (14). vNOTES doesn’t seem to be associated with a higher risk of DVT (14). No DVT or vaginal vault hematoma cases were reported in previous studies reporting SLNB by vNOTES (7–11, 13). Although these infrequently observed intra- and postoperative complications seem not explicitly associated with our surgical technique, more extensive studies are needed to confirm its safety.

We reported a 100% SLN bilateral detection rate. SLNB resulted in a decrease in complications associated with extensive lymphadenectomies while preserving reliable staging results (1). Minimally invasive management of endometrial cancer with SLNB has been increasingly employed with favorable perioperative and long-term outcomes (2, 3, 18). Considering that nearly 40% of the patients with a preoperative diagnosis of endometrial CAH present invasive carcinoma with definitive histology, hysterectomy with the concomitant realization of SLNBs could be a reasonable choice (1, 19). Because pelvic SLNs are no longer identifiable in patients who have undergone a hysterectomy, this approach could lower the number of unnecessary lymphadenectomies (20). For vNOTES SLN mapping, we followed the algorithm proposed by Barlin et al. (21), biopsying any suspicious lymph node other than ICG-fluorescence-positive SLNs. In the case of SLN nonidentification, uni- or bilateral pelvic lymph node dissection could be indicated depending on uterine extemporaneous and definitive pathological results, evaluating the risk of potential nodal involvement (22). Although pelvic lymphadenectomy was never performed in our series, this procedure appears feasible using vNOTES (7, 23). Until now, only transperitoneal vNOTES lymphadenectomies have been reported, but even the retroperitoneal approach appears suitable to dissect all pelvic lymph node stations (8).

vNOTES seems a promising approach for the surgical staging of some gynecological malignancies. In patients with endometrial cancer, vNOTES surgical staging with SLNB appears feasible and potentially advantageous compared to conventional laparoscopy, presenting shorter hospital stays and decreased perioperative morbidities (9). Other potential advantages of a vNOTES approach over conventional laparoscopy are reduced blood loss, less postoperative pain, no complications of abdominal accesses, and better cosmetic results (24, 25). In addition, vNOTES appears safe even for nulliparous women, and patients with previous abdominopelvic surgeries (26, 27). The main limitations are reduced instrument triangulation and restricted space for manipulations, but the use of articulating instruments and adequate training of surgeons can help overcome these difficulties. Because SLNB by vNOTES could be realized with a low CO2 insufflation pressure and no Trendelenburg tilt with reduced impact on cardiorespiratory functions, it appears feasible even for fragile patients frequently encountered in gynecological oncology, such as the elderly and the obese (24, 28). Due to its low surgical impact, vNOTES staging for gynecological malignancies could be performed as an outpatient procedure, as proposed for selected patients operated on by conventional laparoscopy (29). In cervical cancer, SLNB seems as a valuable and safe method to assess the state of lymph nodes in the early-stage cervical cancer (30). Patients with known lymph node metastases are not recommended to undergo radical surgery with lymphadenectomy, and primary chemoradiotherapy should be proposed (31). In an era of increasing use of laparotomy for radical surgeries, preoperative lymph node assessment with a minimally invasive approach appears to reduce the risks associated with unnecessary invasive interventions (1, 32). Despite being reported only once (33), SLNB by vNOTES could be a feasible approach in the staging of early-stage cervical cancers and be used to guide their proper management. The role of vNOTES in ovarian cancer has yet to be defined. Although surgical staging by vNOTES could be feasible in early-stage ovarian cancers (34, 35), some concerns could be associated with its realization, such as the risk of upstaging in the case of ovarian rupture and the risk of understaging due to limited accessibility of some anatomical regions, such as the prevesical peritoneum, costodiaphragmatic recesses, Morrison's pouch, and intestinal mesentery. vNOTES seems an interesting surgical approach for the management of vulnerable cancer patients, for whom one should pay particular attention to postoperative recovery and timing to adjuvant chemo-/radiotherapy. However, strong evidence of its feasibility, the effective perioperative benefits, and the long-term oncological outcomes are needed before expanding the use of vNOTES for the treatment of gynecological malignancies.

A few small studies have reported the use of SLNB in endometrial cancer staging with a transperitoneal vNOTES approach (7, 9, 10, 13). Compared to the largest case series, reported by Wang et al., our study reported similar intraoperative and postoperative outcomes but showed a higher SLN detection rate. They reported an SLN bilateral detection rate of 87% over a total of 23 operated patients, compared to the 100% we observed in our study (9). The SLNs’ detectability could have been influenced by the various surgical approaches. Some authors have raised concerns regarding the transperitoneal approach's difficulty in exposing the caudal part of the pelvic region (9, 10). To solve this problem, Baekelandt firstly described a retroperitoneal vNOTES approach to access pelvic and paraaortic retroperitoneal spaces (8). This approach seems to provide better exposure of the pelvic lymph nodes, and unlike the transperitoneal approach, it allows for the performance of SLNBs as the first surgical step without the need for a previous hysterectomy, expanding its utility to patients with no indication for concomitant hysterectomy (e.g., cervical cancer) and potentially improving the SLNs’ detectability due to earlier lymphatic inspection and no potential artifacts due to fluorescent tracer leakage from the lymph vessels caused by inadvertent manipulations. In endometrial cancer, SLNs are most frequently found on external iliac or obturator locations (2, 4), making their biopsies easy with retroperitoneal vNOTES. However, even SLNs encountered in other locations appeared accessible with this approach (8). Compared to conventional laparoscopy, SLNB by retroperitoneal vNOTES allows inspection of the pelvic retroperitoneal space in a more logical sequence, starting from the cervix and following the lymphatic paths, with a potential improvement in the identification of SLNs. This could be especially important for old or obese patients, for whom there is a greater risk of SLN mapping failure (36, 37).

To the best of our knowledge, SLNB with retroperitoneal vNOTES in endometrial cancer has only been reported twice in the literature (8, 11), and our study represents the first case series focused on this surgical technique. We acknowledge some critical limitations of this study, mainly resulting from its retrospective, single-institution character and the very limited number of patients included in the analyses. These preliminary results are essential, however, for the future development of the vNOTES techniques in gynecological malignancies, but more studies are needed to prove this approach’s feasibility and safety for a larger number of patients.

### Conclusions

Our preliminary study suggests that retroperitoneal vNOTES could be a feasible and safe approach to perform SLNB in endometrial cancer. However, strong evidence of its feasibility, effective benefits, and the long-term oncological outcomes are needed before expanding the use of vNOTES in endometrial cancer outside study settings.

## Data Availability

The raw data supporting the conclusions of this article will be made available by the authors, without undue reservation.
